# Exercise induced stress in horses: Selection of the most stable reference genes for quantitative RT-PCR normalization

**DOI:** 10.1186/1471-2199-9-49

**Published:** 2008-05-19

**Authors:** Katia Cappelli, Michela Felicetti, Stefano Capomaccio, Giacomo Spinsanti, Maurizio Silvestrelli, Andrea Verini Supplizi

**Affiliations:** 1 Department of Pathology, Diagnostic and Veterinary Clinic, University of Perugia, Via San Costanzo 4, 06126 Perugia, Italy; 2 Department of Applied Biology, University of Perugia, Borgo XX giugno 74, 06100 Perugia, Italy; 3 Department of Evolutionary Biology, University of Siena, Via A. Moro 2, 53100 Siena, Italy

## Abstract

**Background:**

Adequate stress response is a critical factor during athlete horses' training and is central to our capacity to obtain better performances while safeguarding animal welfare.

In order to investigate the molecular mechanisms underlying this process, several studies have been conducted that take advantage of microarray and quantitative real-time PCR (qRT-PCR) technologies to analyse the expression of candidate genes involved in the cellular stress response.

Appropriate application of qRT-PCR, however, requires the use of reference genes whose level of expression is not affected by the test, by general physiological conditions or by inter-individual variability.

**Results:**

The expression of nine potential reference genes was evaluated in lymphocytes of ten endurance horses during strenuous exercise. These genes were tested by qRT-PCR and ranked according to the stability of their expression using three different methods (implemented in *geNorm*, *NormFinder *and *BestKeeper*). Succinate dehydrogenase complex subunit A (*SDHA*) and hypoxanthine phosphoribosyltransferase (*HPRT*) always ranked as the two most stably expressed genes. On the other hand, glyceraldehyde-3-phosphate dehydrogenase (*GAPDH*), transferrin receptor (*TFRC*) and ribosomal protein L32 (*RPL32*) were constantly classified as the less reliable controls.

**Conclusion:**

This study underlines the importance of a careful selection of reference genes for qRT-PCR studies of exercise induced stress in horses. Our results, based on different algorithms and analytical procedures, clearly indicate *SDHA *and *HPRT *as the most stable reference genes of our pool.

## Background

Knowledge of the molecular mechanisms underlying the stress response in athlete horse is a fundamental prerequisite for planning an appropriate training schedule to obtain better performances, preserve animal welfare and avoid overtraining-syndrome [1,2].

It is universally accepted that moderate physical activity may have beneficial effects in terms of general health conditions and could favour the functioning of the immune system. Conversely, strenuous exercise, like exhaustive endurance races, may have detrimental effects on the immune system, determine changes in the cellular composition of peripheral blood and induce the expression of genes that appear to be related to the overtraining-syndrome [3-5]. The list of candidate genes is nevertheless far from being complete, as the athlete's reaction to exercise is a coordinated response of multiple organ systems, and likely involves multiple and complex regulatory changes: induction of heat shock proteins, inflammatory response modulation (pro and anti-inflammatory cytokines) and generation of reactive oxygen and nitrogen species (ROS and RSN) that, besides their damaging potential, play a crucial role in cellular signalling [6,7].

Since exercise has been shown to be an important factor in regulating immune cells and their functions, and considering that stress evokes inflammatory reactions, lymphocytes are considered the best candidate cell type to study physiological changes associated with exhaustive exercise [5].

Quantitative real-time PCR (qRT-PCR) is the technique of choice when trying to detect modifications in transcription levels in a reliable and reproducible manner. Nevertheless, there are some technical issues that must be taken into account, such as quality and quantification of the starting material, enzyme efficiency, and primer design. Different approaches have been proposed to normalize measurements of expression levels [8], but this is generally done using an internal control gene, known as a reference gene or as housekeeping gene (HKG), under the assumption that this has a constant level of expression in the chosen tissue, is not affected by the treatment and has no inter-individual variability. In addition, the reference gene and the target gene should have similar ranges of expression to avoid analytical problems.

Widely expressed genes like *ACTB*, *GAPDH *or *R18S *are generally preferred, without preliminary analysis of their expression profiles under the specific study conditions [5,6,9]. Nevertheless, a number of studies report how commonly accepted HKGs do not always constitute reliable controls [10-14], because of unexpected variation in their expression profiles.

More appropriately, multiple HKGs should be evaluated before their employment, and their stability should be measured in the context of the relevant experimental conditions.

A number of statistical methods have been proposed to evaluate stability of gene expression and select the best HKGs in a given experimental setting [9,15-18].

The aim of this paper is to identify the best reference genes for qRT-PCR experiments investigating horse lymphocyte gene expression in exercise induced stress. Statistical algorithms implemented in *geNorm *[9], *BestKeeper *[19], and *NormFinder *[20] were used.

## Results

To assess which are the most stable genes during strenuous exercise, nine potential HKGs were tested in ten endurance horses with a time course sampling strategy. A qRT-PCR assay, based on SYBR^® ^Green detection, was designed for the transcription profiling of the nine genes (*ACTB, B2M, GAPDH, HPRT, R18S*, *RPL32 SDHA, TFRC *and *UBB*, Table 1). The specificity of the amplifications was confirmed by melting curve analyses (Additional files 1, 2, 3, 4, 5, 6, 7, 8, 9). For each assay, a standard curve was generated by using 4-fold serial dilutions of pooled cDNAs. As shown in Table 2, linear correlation coefficients (R^2^) varied from 0.998 to 1.000 and PCR efficiencies (E) ranged between 93.9 and 102.6%.

**Table 1:** Details of the nine genes evaluated.

**Gene Symbol**	**Gene Name**	**Function**	**Accession Number**
*ACTB*	β-actin	Cytoskeletal structural protein	AF035774
*B2M*	β-2-microglobulin	Cytoskeletal protein involved in cell locomotion	X69083
*GAPDH*	glyceraldehyde-3-phosphate dehydrogenase	Glycolytic enzyme	AF157126
*HPRT*	hypoxanthine phosphoribosyltransferase	Metabolic salvage of purines in mammals	AY372182
*R18S*	ribosomal RNA 18S	Member of ribosome RNA	AJ311673
*RPL32*	ribosomal protein L32	Member of ribosomal proteins	CX594263
*SDHA*	succinate dehydrogenase complex subunit A	Electron transporter in the TCA cycle and respiratory chain	DQ402987
*TFRC*	transferrin receptor	Iron uptake	DQ284764
*UBB*	ubiquitin B	Protein degradation	AF506969

**Table 2:** Assay conditions for each of the nine genes evaluated.

**Gene symbol**	**Primers 5'-3' (forward, reverse)**	**Amplicon length, bp**	**PCR efficiency, %**	**Correlation with dilution series (R^2^)**
*ACTB*	GGACCTGACGGACTACCTC CACGCACGATTTCCCTCTC	83	95.2	0.999
*B2M*	CCTGCTCGGGCTACTCTC CATTCTCTGCTGGGTGACG	89	100.2	1.000
*GAPDH*	ATCTGACCTGCCGCCTGGAG CGATGCCTGCTTCACCACCTTC	68	102.6	1.000
*HPRT*	AATTATGGACAGGACTGAACGG ATAATCCAGCAGGTCAGCAAAG	121	93.9	1.000
*R18S*	GTCTGCCCTATCAACTTTCG TTCCTTGGATGTGGTAGCC	119	94.1	0.998
*RPL32*	GGGAGCAATAAGAAAACGAAGC CTTGGAGGAGACATTGTGAGC	138	97.0	1.000
*SDHA*	GAGGAATGGTCTGGAATACTG GCCTCTGCTCCATAAATCG	91	96.0	0.999
*TFRC*	TGGCTACTTGGGCTATTGTAAACG GGTGGTTCTGTTCCCTCTATCTCC	90	97.6	0.998
*UBB*	TTCGTGAAGACCCTGACC CCTTATCCTGGATCTTGGC	91	99.4	0.999

### Expression levels of candidate reference genes

**Figure 1:**
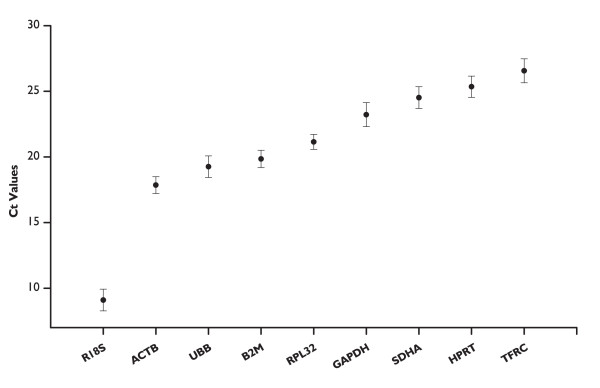
**Average Ct of candidate HKGs**. Expression levels of candidate control genes in the ten endurance horses. Values are given as qRT-PCR cycle threshold numbers (Ct values). Circles represent mean Ct values, bars indicate the standard deviation.

Cycle threshold values (Cts) for the nine HKGs tested ranged between 17.9 (*ACTB*) and 26.6 (*TFRC*). The gene encoding 18S rRNA is largely over expressed (Ct 9.1) compared to the protein coding genes. Each single control gene appeared to be equally expressed in the tested cDNA samples, and the variations of the Ct values (calculated for each single gene in the ten horse individuals subtracting the Min Ct from the Max Ct values) was always smaller than one (Figure 1).

### Data analysis

Profiles obtained for each horse and HKG were analysed using three different methods, implemented in the software *geNorm*, *NormFinder *and *BestKeeper*.

*GeNorm *provides a ranking of the tested genes, based on their expression stability, determining the two most stable HKGs for normalization purposes. Selected HKGs were ranked according to the stability measure M (average pair-wise variation of each gene against all others), from the most stable (lowest M value) to the least stable (highest M value): *SDHA/HPRT*, *R18S*, *B2M*, *UBB*, *ACTB*, *RPL32*, *TFRC*, *GAPDH *(Table 3). All genes displayed a relatively high stability over the three time course samplings, with M values (M < 0.8) far below the accepted limit of 1.5 [9]. The two most stably expressed genes of our pool (*SDHA *and *HPRT*) allow an optimal normalization of qRT-PCR data, and the addition of a third HKG (*R18S*) would not significantly increase the statistical reliability of this calculation (V_2/3 _= 0.090, abundantly below the default cut-off value of 0.15 [9]).

**Table 3:** Candidate reference genes ranking according to *geNorm*.

***Rank***	***Gene Symbol***	***M value***	***V value***
1/2	*SDHA/HPRT*	0.232	---
3	*R18S*	0.274	0.090
4	*B2M*	0.311	0.078
5	*UBB*	0.335	0.064
6	*ACTB*	0.376	0.068
7	*RPL32*	0.405	0.059
8	*TFRC*	0.448	0.065
9	*GAPDH*	0.521	0.080

M value is calculated as average expression stability of control genes during stepwise exclusion of the least stable controls. V value is calculated as the pair-wise variation between two sequential normalization.

The *NormFinder *algorithm uses a model-based approach for the estimation of modifications among the HKG expressions, also taking into account variation across sub-groups and avoiding artificial selection of co-regulated genes [20]. The results of the *NormFinder *analysis are shown in Table 4. This ranking appeared to be slightly different from what obtained using *geNorm*. GAPDH, TFRC and RPL32 still occupy the lowest positions, while SDHA remains the most stable gene. ACTB gained the second position stepping over HPRT and R18S defined as the least reliable controls.

**Table 4:** Candidate reference genes ranking according to *NormFinder*.

***Rank***	***Gene Symbol***	***Stability value***
1	*SDHA*	0.056
2	*ACTB*	0.086
3	*R18S*	0.087
4	*HPRT*	0.093
5	*B2M*	0.103
6	*UBB*	0.111
7	*RPL32*	0.124
8	*TFRC*	0.151
9	*GAPDH*	0.174

*BestKeeper *measures HKG stability by using a pair-wise correlation analysis of all pairs of candidate genes and calculating the geometric mean of the best candidates [15,19]. A preliminary analysis, based on the inspection of raw Ct values, estimated the variation of all HKGs to be compatible with an overall stability in gene expression (Table 5), with SD values lower than 1. All genes were retained for the calculation of the *BestKeeper *index, which similarly exhibited a moderate SD variation (0.58). *BestKeeper *allows a comparative analysis across HKGs, by estimating correlations in the expression levels between all the possible candidates. Highly correlated control genes are combined into an index. Afterwards, the pair-wise correlation between genes and the correlation between each gene and the index are calculated, describing the consistency between the index and each HKG [15].

**Table 5:** Statistical output from *BestKeeper *analysis.

	*ACTB*	*B2M*	*GAPDH*	*HPRT*	*R18S*	*RPL32*	*SDHA*	*TFRC*	*UBB*
N	30	30	30	30	30	30	30	30	30
G Mean [Ct]	17.85	19.84	23.20	25.34	9.07	21.15	24.51	26.54	19.25
A Mean [Ct]	17.86	19.85	23.22	25.35	9.10	21.15	24.52	26.56	19.26
Min [Ct]	16.92	18.55	20.99	24.11	7.97	20.10	23.22	25.11	17.94
Max [Ct]	19.23	21.86	25.05	27.76	12.02	22.42	26.91	28.88	21.46
SD [± Ct]	0.49	0.51	0.71	0.62	0.57	0.46	0.66	0.73	0.64
CV [% Ct]	2.77	2.57	3.08	2.43	6.28	2.18	2.70	2.75	3.35
coeff. of corr. [r]	0.890	0.921	0.708	0.963*	0.949*	0.832	0.983*	0.844	0.935*
p-value	0.001	0.001	0.001	0.001	0.001	0.001	0.001	0.001	0.001

Genes are listed in alphabetical order. N: number of samples; G Mean [Ct]: geometric mean of the Ct; A Mean [Ct]: arithmetic mean of the Ct; Min and Max [Ct]: extreme values of the Ct; SD [Ct]: standard deviation of the Ct; CV [% Ct]: coefficient of variance expressed as percentage on the Ct level. * Indicates the best correlation between control genes and the *BestKeeper *index.

The nine control genes tested in our analysis correlated well one with one another and with the *BestKeeper *index (Table 5). The best correlation between one HKG and the *BestKeeper *index was obtained for *SDHA *(r = 0.983), followed by *HPRT, R18S *and *UBB*. The statistically significant correlation shown by *SDHA *with the *BestKeeper *index appeared to be consistent with the good performance of this gene as assessed by *geNorm *and *NormFinder*. It is also remarkable how *GAPDH*, *RPL32 *and *TFRC*, are again classified as the least reliable HKGs, showing the worst correlations with the determined *BestKeeper *index (Table 5).

## Discussion

A number of authors have studied gene expression profiles in exercise induced stress using forefront technologies, like gene chips and qRT-PCR. This study is the first solid contribution in assessing which reference genes have to be used to validate and normalize qRT-PCR outcomes.

Several methods have been proposed to allow accurate normalization of gene expression using qRT-PCR [9,19-22] but at present there is no consensus on which algorithm should be used to measure reference gene stability. A comparison of different methods of reference gene selection allows a better identification of the most reliable controls and reduces the risk of artificial selection of co-regulated transcripts [16].

We compared three different statistical approaches (*geNorm*, *NormFinder *and *BestKeeper*) to evaluate nine potential HKGs, in order to select the best reference gene to be used in studying exercise-induced stress in horses.

The uniformity in gene ranking between the three software packages was generally high: *SDHA *is the most stable HKG according to all the three methods. *HPRT *similarly displays a constant significant stability. *R18S *always ranks third, *B2M *fourth and *UBB *fifth, and can be therefore considered plausible HKGs, even if the addition of supplementary reference genes would not significantly enhance the reliability of the normalization according to the *geNorm *analysis (V value, Table 3).

Regarding *ACTB*, it is difficult to formulate a final judgement because, as already reported in a previous study [15], its classification is not consistent between the three software packages (6^th ^in *geNorm *and *BestKeeper*, 2^nd ^in *NormFinder*). Nevertheless, this gene shows an overall reduced variability, as attested by the M value calculated by *geNorm *and by its good correlation with the *BestKeeper *index (r = 0.890).

*GAPDH*, *TFRC *and *RPL32 *were classified as the least stable genes and they are not likely to be useful in this given experimental system. Notably, the expression of *GAPDH*, that has been used as HKG in a previous exercise induced stress study [5], appears to be the least stable.

In contrast with what reported elsewhere [23-26], *R18S *appears to be a good potential reference gene. Despite its good performance, the usefulness of this gene as a control is often doubted: some authors [27] tend to consider it unsuitable for normalization because its transcription is carried out by RNA polymerase I and because of its well known over-expression in comparison with mRNAs (as confirmed even in our experiments, Figure 1). Considering that HKGs that have expression levels comparable to the gene of interest are generally preferred [15], its usage should be carefully considered if used for the normalization of genes that exhibit low level of expression.

## Conclusion

Our results indicate *SDHA *and *HPRT *as the most stable reference genes with a very good statistical reliability according to all the three software employed. Moreover, the use of only two genes (*SDHA *and *HPRT*) appears to be sufficient for a reliable normalization of the genes of interest; this result is of special interest for future high throughput applications of the technique.

## Methods

### Blood collection, RNA extraction and cDNA synthesis

Ten horses were chosen among participants to national endurance races (90–120 km). Blood samples were taken from the jugular vein and collected at three different time points: before, at the end of the race, and 24 hours after the race. Immediately after collection, peripheral blood mononuclear cells (PBMCs) were isolated by the Ficoll-Hypaque method (GE Healthcare, Pollards Wood, United Kingdom) from 8 ml of blood. Total RNA was extracted from approximately 1 × 10^7 ^PBMCs, using the Aurum Total RNA Fatty and Fibrous Tissue kit (Bio-Rad, Herculers CA, USA) according to the manufacturer's instructions. Genomic DNA was eliminated by a DNase treatment supplied with the kit. Extracted RNA was quantified using the Quant-It RNA assay (Invitrogen, Dorset, United Kingdom) in a VersaFluor fluorometer (Bio-Rad) and checked for integrity in a denaturing agarose gel electrophoresis with ethidium bromide staining. Successful removal of DNA contaminants was tested by absence of PCR amplification in the *MC1R *gene (GenBank accession number X98012, primers from [28]). 1.5 μg of total RNA were retro-transcribed using random hexamers and Superscript III Reverse Transcriptase (Invitrogen) according to the manufacturer's specifications. A PCR with *ACTB *primers (Table 2) was performed on each cDNA to check for successful retro-transcription.

### Reference genes selection and primer design

Nine widely used reference genes were evaluated: β-actin (*ACTB*), glyceraldehyde-3P-dehydrogenase (*GAPDH*), hypoxanthine ribosyltransferase (*HPRT1*), β-2-microglobin (*B2M*), succinate dehydrogenase complex subunit A (*SDHA*), transferrin receptor (*TFRC*), ubiquitin B (*UBB*), ribosomal protein L32 (*RPL32*) and 18S ribosomal rRNA (*R18S*). In order to minimize the possibility of co-regulation, genes were selected from different functional classes.

Primers were designed based on available sequences using the Primer3 software.

Mfold [29] was used to check the chosen sequences to avoid designing primers in the region of template secondary structure; amplicon lengths were optimized to 68/138 bp to ensure optimal polymerization efficiency. Specificity of amplification was confirmed by sequencing.

For each primer pair, a preliminary real-time assay was performed to evaluate the amplification of non-specific products or primer dimer artefacts (Additional files 1, 2, 3, 4, 5, 6, 7, 8, 9). Efficiency of RT-PCR (E), slope values, and correlation coefficients (R^2^) were determined (Table 2) using serial 1:4 dilutions of a template cDNA (pooled from the ten individuals studied, previously diluted 1:5).

PCR products were subsequently resolved on 2% agarose gel to check for size specificity of the amplicon.

### Real-time quantitative PCR

Five microliters of cDNA template (previously diluted 1:10) were added to the master mix FastStart SYBR Green Master (Roche Applied Science, Penzberg, Germany) with the ROX fluorochrome internal check. PCR reactions, in a volume of 25 μl were performed on a MX3000P machine (Stratagene, La Jolla CA, USA). PCR conditions were the same for all primer pairs: initial denaturation at 95°C for 10' followed by 40 cycles of denaturation at 95°C for 30", annealing at 58°C for 30" and extension at 72°C for 30". Fluorescence data were collected at the end of the extension step. Following cycling, the melting curve was determined in the range 58°–95°C, with a temperature slope of 0.01°C/sec. Each reaction was run in triplicate with appropriate negative controls.

Baseline and threshold values were automatically determined for all plates and genes using the MxPro software ver. 3.20 (Stratagene). In order to ensure comparability between data obtained from different experimental plates, threshold values for each gene were manually set to the arithmetic mean between the thresholds as automatically determined following each run. Corrected Ct values were transformed to quantities based on the comparative Ct method. Following appropriate formatting, values were imported into *geNorm *(version 3.4), *NormFinder *(version 0.953) and *BestKeeper *(version 1) VBA applets.

## Authors' contributions

KC, MF, SC performed all experiments and data analysis, and drafted the manuscript. GS supervised the study design and the data analysis. MS conceived the project, AVS supervised and coordinated the project and participated in writing the manuscript. All authors read and approved the final manuscript.

## Supplementary Material

Additional file 1 **Melting curve *ACTB *gene**. Melting curve analyses image (jpg format) collected using using the MxPro software ver. 3.20 (Stratagene) during calibration experiments of the selected primer pair for the *ACTB *gene. Click here for file

Additional file 2 **Melting curve *B2M *gene**. Melting curve analyses image (jpg format) collected using using the MxPro software ver. 3.20 (Stratagene) during calibration experiments of the selected primer pair for the *B2M *gene. Click here for file

Additional file 3 **Melting curve *GAPDH *gene**. Melting curve analyses image (jpg format) collected using using the MxPro software ver. 3.20 (Stratagene) during calibration experiments of the selected primer pair for the *GAPDH *gene. Click here for file

Additional file 4 **Melting curve *HPRT *gene**. Melting curve analyses image (jpg format) collected using using the MxPro software ver. 3.20 (Stratagene) during calibration experiments of the selected primer pair for the *HPRT *gene. Click here for file

Additional file 5 **Melting curve *R18S *gene**. Melting curve analyses image (jpg format) collected using using the MxPro software ver. 3.20 (Stratagene) during calibration experiments of the selected primer pair for the *R18S *gene. Click here for file

Additional file 6 **Melting curve *RPL32 *gene**. Melting curve analyses image (jpg format) collected using using the MxPro software ver. 3.20 (Stratagene) during calibration experiments of the selected primer pair for the *RPL32 *gene. Click here for file

Additional file 7 **Melting curve *SDHA *gene**. Melting curve analyses image (jpg format) collected using using the MxPro software ver. 3.20 (Stratagene) during calibration experiments of the selected primer pair for the *SDHA *gene. Click here for file

Additional file 8 **Melting curve *TFRC *gene**. Melting curve analyses image (jpg format) collected using using the MxPro software ver. 3.20 (Stratagene) during calibration experiments of the selected primer pair for the *TFRC *gene. Click here for file

Additional file 9 **Melting curve *UBB *gene**. Melting curve analyses image (jpg format) collected using using the MxPro software ver. 3.20 (Stratagene) during calibration experiments of the selected primer pair for the *UBB *gene. Click here for file
