# Supplementary figures and images for: Exercise induced stress in horses: Selection of the most stable reference genes for quantitative RT-PCR normalization
Source: BMC Mol Biol. 2008 May 19;9:49. doi: 10.1186/1471-2199-9-49 (PMC2412902; doi:10.1186/1471-2199-9-49)

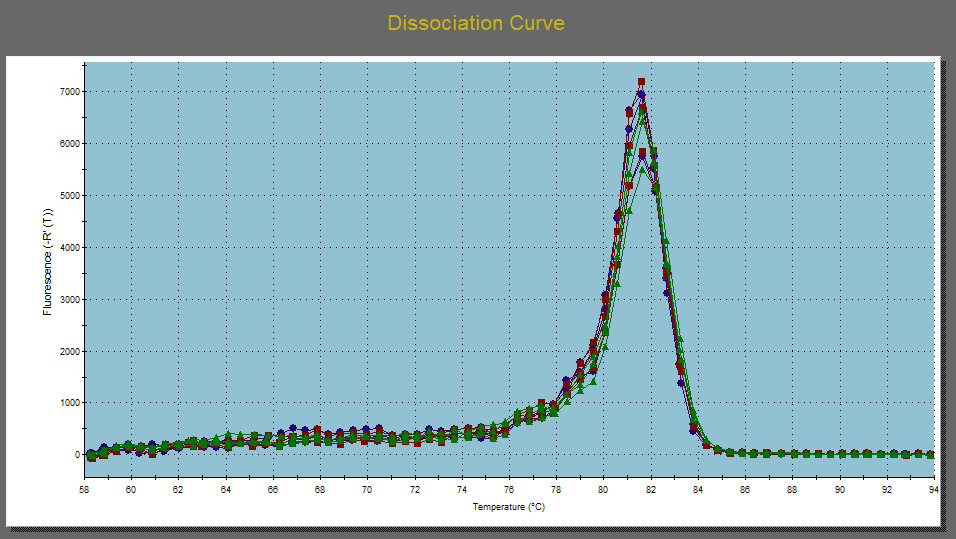

Supplement: Additional file 1 — Melting curve ACTB gene. Melting curve analyses image (jpg format) collected using using the MxPro software ver. 3.20 (Stratagene) during calibration experiments of the selected primer pair for the ACTB gene. [file 1471-2199-9-49-S1.jpeg]

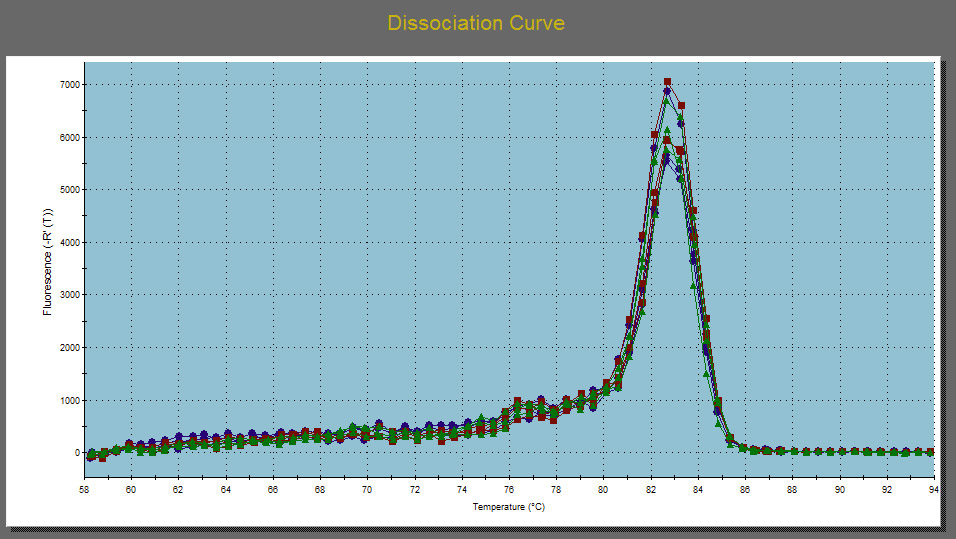

Supplement: Additional file 2 — Melting curve B2M gene. Melting curve analyses image (jpg format) collected using using the MxPro software ver. 3.20 (Stratagene) during calibration experiments of the selected primer pair for the B2M gene. [file 1471-2199-9-49-S2.jpeg]

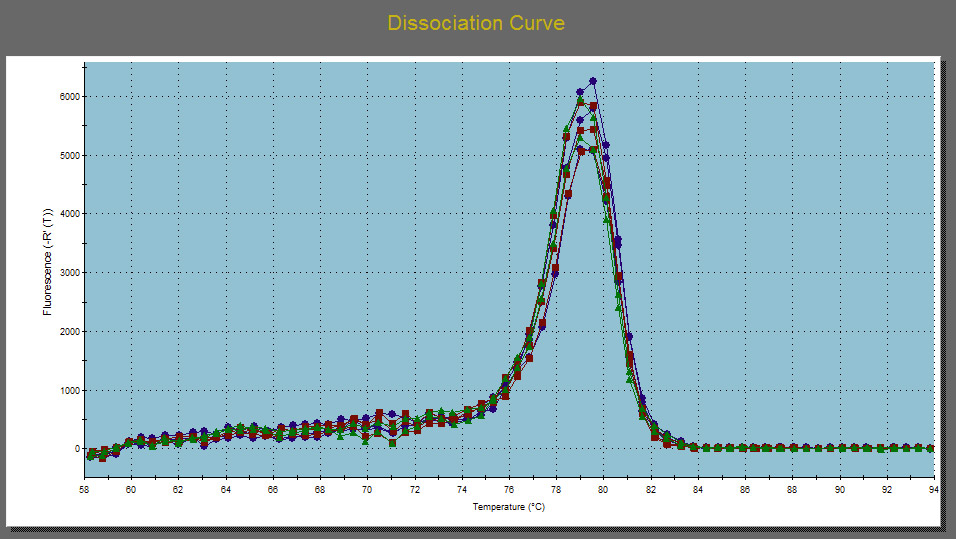

Supplement: Additional file 3 — Melting curve GAPDH gene. Melting curve analyses image (jpg format) collected using using the MxPro software ver. 3.20 (Stratagene) during calibration experiments of the selected primer pair for the GAPDH gene. [file 1471-2199-9-49-S3.jpeg]

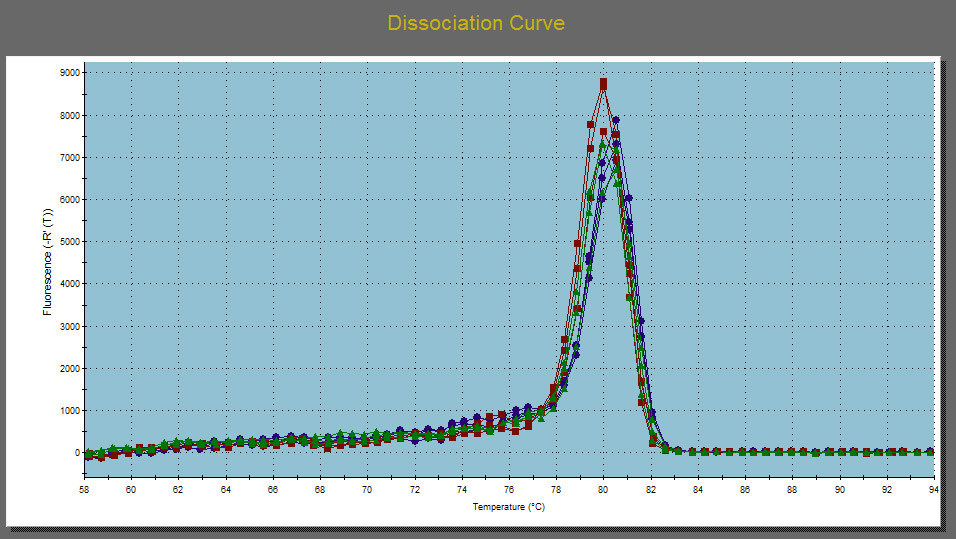

Supplement: Additional file 4 — Melting curve HPRT gene. Melting curve analyses image (jpg format) collected using using the MxPro software ver. 3.20 (Stratagene) during calibration experiments of the selected primer pair for the HPRT gene. [file 1471-2199-9-49-S4.jpeg]

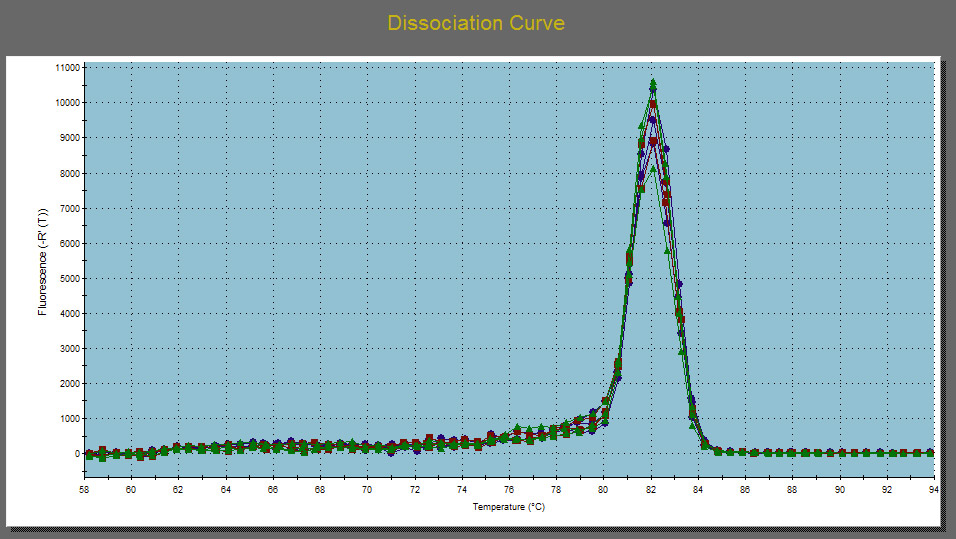

Supplement: Additional file 5 — Melting curve R18S gene. Melting curve analyses image (jpg format) collected using using the MxPro software ver. 3.20 (Stratagene) during calibration experiments of the selected primer pair for the R18S gene. [file 1471-2199-9-49-S5.jpeg]

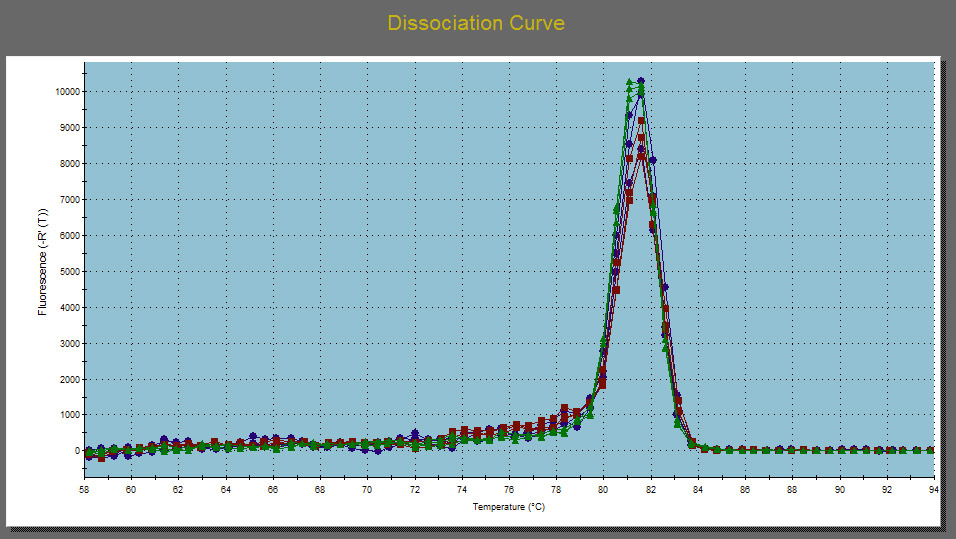

Supplement: Additional file 6 — Melting curve RPL32 gene. Melting curve analyses image (jpg format) collected using using the MxPro software ver. 3.20 (Stratagene) during calibration experiments of the selected primer pair for the RPL32 gene. [file 1471-2199-9-49-S6.jpeg]

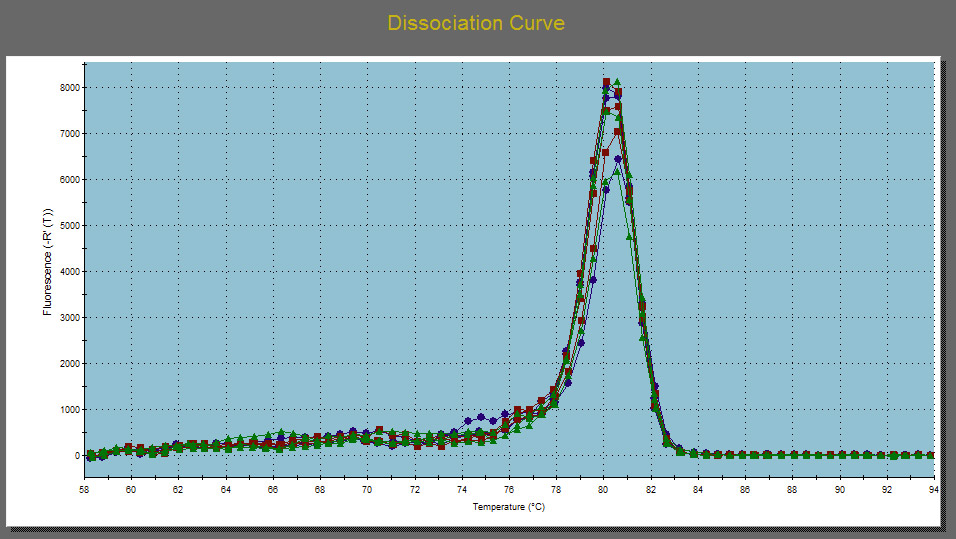

Supplement: Additional file 7 — Melting curve SDHA gene. Melting curve analyses image (jpg format) collected using using the MxPro software ver. 3.20 (Stratagene) during calibration experiments of the selected primer pair for the SDHA gene. [file 1471-2199-9-49-S7.jpeg]

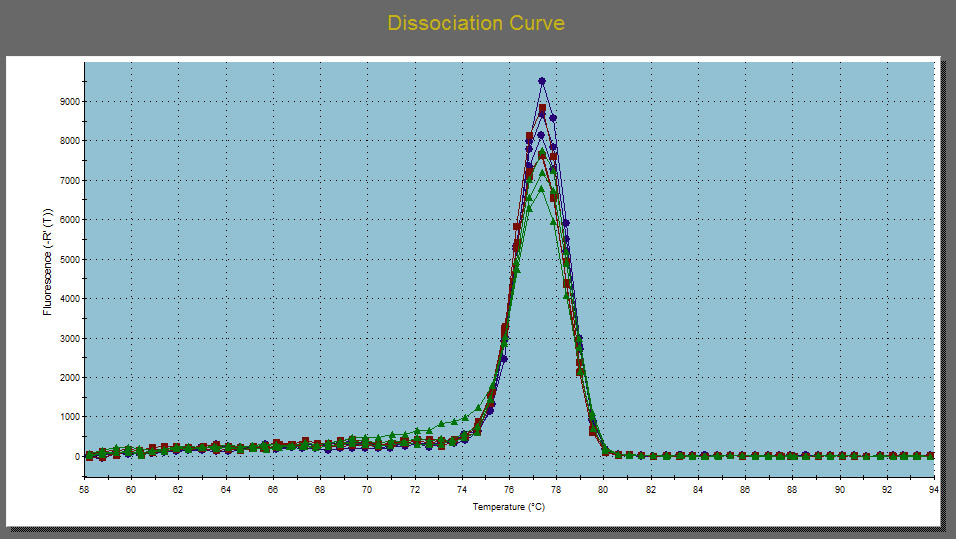

Supplement: Additional file 8 — Melting curve TFRC gene. Melting curve analyses image (jpg format) collected using using the MxPro software ver. 3.20 (Stratagene) during calibration experiments of the selected primer pair for the TFRC gene. [file 1471-2199-9-49-S8.jpeg]

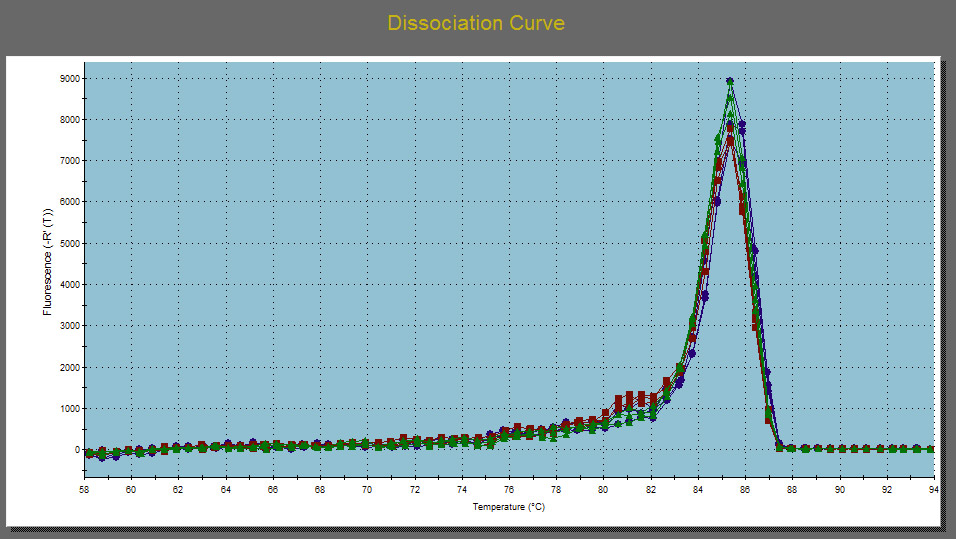

Supplement: Additional file 9 — Melting curve UBB gene. Melting curve analyses image (jpg format) collected using using the MxPro software ver. 3.20 (Stratagene) during calibration experiments of the selected primer pair for the UBB gene. [file 1471-2199-9-49-S9.jpeg]
